# HIF-α Prolyl Hydroxylase Inhibitors and Their Implications for Biomedicine: A Comprehensive Review

**DOI:** 10.3390/biomedicines9050468

**Published:** 2021-04-24

**Authors:** Kiichi Hirota

**Affiliations:** Department of Human Stress Response Science, Institute of Biomedical Science, Kansai Medical University, Hirakata, Osaka 573-1010, Japan; khirota-kyt@umin.ac.jp; Tel.: +81-72-804-2526

**Keywords:** hypoxia, transcription factor, hypoxia-inducible factor 1, HIF-1, hypoxia sensing, HIF-PH inhibitor

## Abstract

Oxygen is essential for the maintenance of the body. Living organisms have evolved systems to secure an oxygen environment to be proper. Hypoxia-inducible factor (HIF) plays an essential role in this process; it is a transcription factor that mediates erythropoietin (EPO) induction at the transcriptional level under hypoxic environment. After successful cDNA cloning in 1995, a line of studies were conducted for elucidating the molecular mechanism of HIF activation in response to hypoxia. In 2001, cDNA cloning of dioxygenases acting on prolines and asparagine residues, which play essential roles in this process, was reported. HIF-prolyl hydroxylases (PHs) are molecules that constitute the core molecular mechanism of detecting a decrease in the partial pressure of oxygen, or hypoxia, in the cells; they can be called oxygen sensors. In this review, I discuss the process of molecular cloning of HIF and HIF-PH, which explains hypoxia-induced EPO expression; the development of HIF-PH inhibitors that artificially or exogenously activate HIF by inhibiting HIF-PH; and the significance and implications of medical intervention using HIF-PH inhibitors.

## 1. Introduction

Chronic kidney disease (CKD), cancers, inflammatory diseases, nutritional deficiencies, genetic disorders, and drugs may cause anemia. Erythropoiesis-stimulating agents (ESAs), including recombinant erythropoietin (EPO) analogs, have been used for treating anemia associated with CKD through compensating for decreased EPO [1,2,3]. However, resistance and tolerance to these drugs have been shown to develop, and high doses of ESAs can cause side effects such as cardiovascular diseases [4].

Anemia is a condition in which the blood is unable to deliver sufficient amounts of oxygen to the tissues because of insufficient number of red blood cells (RBCs), which are the oxygen carriers in the blood, and insufficient hemoglobin, which holds molecular oxygen in the RBCs [5,6,7]. Oxygen is essential for the maintenance of the body [8]. Since there is no biosynthetic system for oxygen in the body, the supply of oxygen to the organism depends solely on the supply from the outside world, usually through gas exchange in the lungs. For oxygen to be distributed throughout the body in this manner, blood vessels must be lined throughout the body, RBCs must be produced appropriately, oxygen must be delivered in a hemoglobin-bound state to the cells that require oxygen, and the transported oxygen must be consumed appropriately [9,10]. Living organisms have evolutionarily constructed the systems for the purpose [8,10]. Hypoxia-inducible factor (HIF) plays an essential role in this process; it is a transcription factor that was genetically isolated while elucidating the molecular mechanism through which EPO is induced at the transcriptional level in hypoxia [11]. After successful cDNA cloning in 1995 [12,13,14], studies were conducted to elucidate the molecular mechanism through which HIF is activated in response to hypoxia. In 2001, the cloning of dioxygenases or hydroxylases acting on proline and asparagine residues, which play an important role in this process, was reported [15,16,17,18]. In particular, the enzyme that hydroxylates the proline residues of the HIF-α subunit is a dioxygenase or hydroxlase whose substrates are the HIF-α subunit, α-ketoglutarate (2-oxoglutarate: 2-OG), and molecular oxygen (O_2_); its cofactors are divalent iron (Fe^2+^) and ascorbic acid. Thus, HIF-prolyl hydroxylases (HIF-PHs) constitute the core molecular mechanism of detecting a decrease in the partial pressure of oxygen, or hypoxia, in the cells; they can be called oxygen sensors [19,20].

Various strategies have been developed for inducing HIF activation under normoxic conditions, such as chelation of divalent iron and cobalt ion treatment. Certain 2-OG analogs have been reported to activate HIF. Pharmacological inhibition of HIF-PHs increases EPO and DMT1 (ferrous ion membrane transport protein 1)/Nramp2 (NRAMP metal ion transporter 2) in the intestinal epithelium and decreases hepcidin production in the liver, thereby improving iron metabolism in vivo and potentially leading to efficient treatment of not only CDK-induced anemia but also anemia associated with chronic diseases [11,21,22,23].

In this review, I describe the process of molecular cloning of HIF and HIF-PH, which explains the induction of EPO expression by hypoxia; the development of HIF-PH inhibitors (HIF-PHIs) that artificially activate HIF by inhibiting HIF-PH; medical interventions that utilize HIF-PHIs; and the implications and significance of these interventions [24,25]. In addition, this review will specifically describe HIF-PHIs, which are currently prescribed in medical practice, and explain its biological effects in addition to its anemia-alleviating effects in relation to its molecular mechanism.

## 2. Sensing of Hypoxia and Execution of the Hypoxic Gene Responses

The body has evolved systems for monitoring oxygen concentration in various organs and tissues and for responding to deviations in oxygen partial pressure. The hypoxic response of living organisms is diverse and multilayered, reflecting the fact that oxygen is essential for life support [8,26].

There is a system that signals a decrease in the partial pressure of arterial blood oxygen to the respiratory center in brainstem via carotid body-glossopharyngeal nerve and aortic body-vagus nerve [27]. In this system, glomus type I cells in the carotid body serve as hypoxia sensor cells. In addition, small pulmonary arteries contract in response to alveolar hypoxia (hypoxic pulmonary vasoconstriction) [28]. In this case, the alveolar epithelium may act as hypoxia sensor cells. Thus, these hypoxic responses act through specific mechanisms for sensing hypoxia. Hypoxic signaling in glomus type I cells and the pulmonary artery smooth muscle is mediated by potassium channel-calcium channel coupling. However, the molecular mechanisms through which hypoxic signaling is mediated by these channels remain unclear. The study of hypoxic response has been preceded by electrophysiological studies, which included the above-mentioned study on the involvement of ion channels. Studies using classical biochemical methods have a long history; however, molecular biology methods were not introduced until the 1990s. An important turning point in this research was the isolation of the gene for HIF-1, which was identified as a transcription factor involved in the hypoxic induction of EPO [20,29].

## 3. Exploration of the Mechanism of Erythropoietin (EPO) Production Induction

In the early 1990s, it was assumed that circulating factors stimulating hematopoiesis were present in the serum of phlebotomized rabbits, as demonstrated by parabiosis experiments in rats in 1950 [30]. The name erythropoietin was proposed for this hypothetical liquid factor; however, the molecular mechanism underlying its induction was unknown. At that time, this factor was hypothesized to be secreted by the pituitary gland. In 1957, EPO was shown to be produced by the kidneys [31,32,33]. In 1977, Miyake and his colleagues succeeded in purifying EPO from a large amount of urine from a patient with aplastic anemia [34]. EPO acts on the erythroid progenitor cells in the bone marrow to promote their differentiation and proliferation into RBCs. In 1985, EPO gene cloning was successfully developed [35], paving the way for the production of large amounts of EPO through genetic engineering. Additionally, approximately 30 years ago, a therapeutic method was established for administering EPO produced exogenously through genetic engineering.

Until recently, there was debate regarding the sites and cells involved in EPO production. The candidates included the juxtaglomerular apparatus, proximal tubules of the kidney, and vascular endothelial cells. Finally, an elegant analysis using genetically modified mice revealed tubulointerstitial cells in the kidney. There is a consensus that EPO secreted into the bloodstream is produced primarily by renal EPO-producing (REP) cells, with supplementary production in the liver. Although there are reports of EPO production in the brain, it is believed that EPO acts as a paracrine factor rather than being secreted into the circulating blood [36].

In recent years, therapeutic approaches have emerged that induce EPO production in vivo through the administration of exogenous small-molecule compounds, namely HIF-prolyl hydroxylase inhibitors (PHIs); five HIF-PHI agents are available for prescription in clinical practice in Japan.

Research on the regulation of EPO expression is interlinked with research on hypoxia-induced gene responses. Erythropoiesis has been observed to be stimulated in hypoxic environments. This phenomenon can be applied to sports medicine through high-altitude training. The increase in RBCs, hemoglobin, and blood volume improves the body’s ability to carry more oxygen in the blood, leading to an increase in endurance (aerobic exercise capacity). Effective utilization of oxygen by the muscles is improved by the development of skeletal muscle capillaries and increased concentrations of the oxygen storage protein myoglobin, as well as the induction of enzymes involved in oxidative phosphorylation in the mitochondria and increased mitochondrial mass [37]. This a topic that needs further research as there are conflicting observations with regard to the effectiveness of high-altitude training.

Research on the molecular biology of hypoxia-induced gene response began in the late 1980s. In 1987, a research group at Harvard University demonstrated that a human hepatocarcinoma-derived established cell line produced EPO in response to hypoxia [38]. They found that not only hypoxia but also iron chelators and CoCl_2_ stimulate EPO production in cells [38,39,40].

Thus, the modern era of research on the biology of hypoxia-induced gene responses began with the discovery that EPO, which had been believed to be produced by specialized cells in the kidney stroma, could also be produced by established cell lines that could be grown indefinitely on a Petri dish. In other words, molecular biological methods could be adapted to this research.

## 4. Molecular Cloning of Hypoxia-Inducible Factor 1

Eight years later, a transcription factor involved in EPO induction under hypoxic conditions was isolated. The protein and cDNA of hypoxia-inducible factor 1 was purified and isolated in 1995 using a method in which a protein that binds to a specific DNA sequence (hypoxia response element; HRE; 5′-R(A/G)CGTG-3′) on the EPO gene was purified from established cell line HeLa cells [12,13,14].

HIF-1 is a transcription factor consisting of an α subunit (HIF-1α) and a β subunit (HIF-1β) with helix-loop-helix (HLH) and Per-ARNT-SIM (PAS) domains, which are hydrophobically linked to each other to form a functional protein [12]. The activated and dimerized HIF-1 (HIF-1α/HIF-1β) translocates to the cell nucleus and binds to the HRE in the regulatory region of the target gene to promote the expression of the dominant gene. The expression of the HIF-1α protein is maintained to be very low in culture under 20% oxygen conditions; however, it increases rapidly in response to a decrease in oxygen partial pressure below 5% oxygen conditions [41]. In addition, the activity of HIF as a transcription factor increases with decreasing oxygen partial pressure, independent of the change in protein expression [18]. In this scheme, HIF-1α accumulated in the cell forms a heterodimer with HIF-1β, and the activated HIF-1 moves into the cell nucleus, where it binds to the expression control region and promotes the expression of the target gene [20,29].

HIF-1α, which was isolated in this manner, has been found to have the HIF-2α and HIF-3α family of molecules [42]. In particular, HIF-2α is highly homologous to HIF-1α; however, there are certain dissimilarities in hypoxia-inducible gene expression. As discussed later, several genes that are involved in iron metabolism are regulated by HIF-2 rather than HIF-1 [42]. Surprisingly, although HIF-1 was isolated as a factor responsible for hypoxia-induced expression of EPO, it is HIF-2, not HIF-1, that is responsible for hypoxia-induced expression of EPO in the kidney [43,44].

## 5. Intracellular Signaling Pathways Linking Hypoxia and HIF Activation

Following the cDNA cloning of HIF-1 [13], the molecular mechanism through which HIF activity is increased under hypoxic conditions remained a major question, the solution to which was expected to resolve the larger problem of how cells sense and signal a decrease in the partial pressure of oxygen.

A major turning point in the elucidation of this mechanism was the discovery that the HIF-1α protein is ubiquitinated in the presence of ample oxygen and is consequently sequestered to the proteasome for degradation [45,46]. The VHL protein, which exhibits E3 ubiquitin ligase activity, plays a central role in HIF-1α ubiquitination. The VHL gene has been identified as the causative gene in von Hippel–Lindau disease. It is a tumor suppressor gene, and the deficiency of this gene causes hemangioblastoma of the brain and retina and renal cell carcinoma [47,48,49,50]. The VHL protein is a component of a protein complex that includes elongin B, elongin C, and cullin-2. Nevertheless, the molecular mechanism of HIF-1α ubiquitination, caused by the decrease in oxygen partial pressure, was unclear.

Under conditions of sufficient oxygen, the newly translated HIF-1α protein from the mRNA undergoes hydroxylation at the proline residues, which results in hydroxylated-proline residue-dependent ubiquitination by the VHL system and transport to the proteasome, the intracellular protein destruction machinery, for degradation [51,52]. HIF-1α and its family member HIF-2α are regulated in a similar manner [53,54]. However, it was reported that the transcriptional activity of HIF-1α and HIF-2α is regulated by the hydroxylation of asparagine residues, which are conserved across species in the transcriptional domain of HIF-1α, in addition to protein stabilization [18]. In the hydroxylated state, association with basic transcription factors such as p300 is inhibited, and sufficient transcriptional activity is not achieved. Exposure to hypoxia decreases the hydroxylation of asparagine residues and renews transcriptional activity.

**Figure 1:**
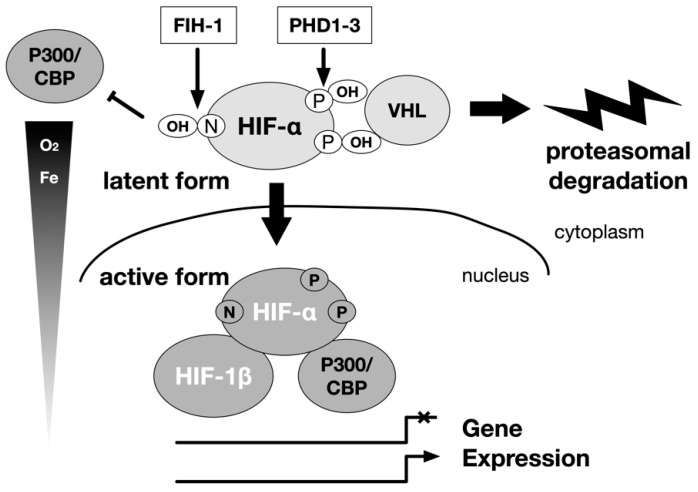
Regulation of α subunits of HIF-1 and HIF-2 by hydroxylation. The main stream of HIF-1 and HIF-2 activation is carried out by the hydroxylase of the HIF-α subunit. The hydroxylation is carried out by the prolyl hydroxylase domain (PHD) protein and the factor inhibiting HIF-1 (FIH-1) protein. Oxygen is the substrate of these enzymes. A decrease in the concentration of the substrate oxygen leads to a decrease in the hydroxylation reaction, and the HIF-1α and HIF-2α are spared from destruction in the proteasome. Then, the HIF-α protein accumulates in the cell, becomes active as a transcription factor. They form a heterodimer with the HIF-1β subunit, and translocate from the cytoplasm to the nucleus to regulate gene expression. The intracellular elements that affect this reaction can be regulators of HIF-1 activity independent of oxygen partial pressure. P: proline, N: asparagine.

In other words, under hypoxia, the hydroxylation of proline and asparagine residues is inhibited, protein destruction is reduced, and HIF-1α accumulates in the cell, allowing it to associate with basic transcription factors such as p300, leading to activation (Figure 1).

In 2001, results were published regarding molecular cloning of oxygenase (dioxygenase) using 2-OG, molecular oxygen, and the HIF-1 protein as the substrate for hydroxylation [15,16,17].

## 6. Genetic Cloning of Enzymes Modifying HIF-α Hydroxylation (PHDs and FIH-1)

**Figure 2:**
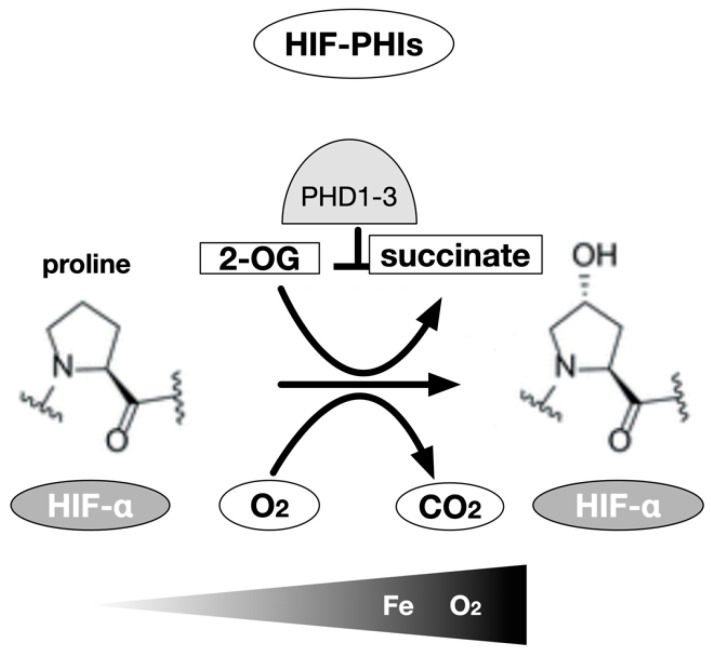
HIF-α prolyl hydroxylase. The hydroxylation of proline residues is an enzymatic reaction using 2-oxoglutarate as a substrate in addition to HIF-1α or HIF-2α subunits and molecular oxygen. Three types of prolyl hydroxylase domains are known in humans, but in most cells, PHD2 is mainly involved. Theoretically, chelation of the coenzyme Fe (II) also inhibits the activity of the enzyme, but the inhibitors currently used in clinical medicine work in competition with 2-OG to inhibit this enzyme.

The cDNAs of the oxygenases were isolated. Three isozymes of proline hydroxylase were designated as prolyl hydroxylase domain (PHD)1-3 [15] and an asparagine residue hydroxylase as a factor inhibiting HIF (FIH)-1 [16,17]. The regulation of intracellular accumulation of HIF-α protein including HIF-1α and HIF-2α and the TAD (trans activational domain) transcriptional activity of HIF-α by PHD and FIH-1, respectively, are mediated by the hydroxylation of amino acid residues [17]. It has been proposed that the enzymatic activity of PHD decreases in response to a decrease in oxygen concentration and the protein expression of HIF-α increases. Subsequently, when the oxygen concentration decreases further, the enzymatic activity of FIH-1 decreases, p300 binds to TAD, and the transcriptional activity of HIF increases to its maximum activity [17,18]. Three PHD genes have been identified in mammals, and each gene product is believed to perform a specific function, as they differ with respect to the organ of expression and subcellular localization [15,55,56,57]. In vitro experiments have shown that all three PHD genes hydroxylate specific proline residues of HIF-α [55]. In in vivo conditions, however, PHD2 is the major proline hydroxylase for HIF-α; it has been shown to be essential for biogenesis through gene disruption experiments [55]. PHD1 and PHD2 also negatively regulats the HIF-mediated hypoxia response by hydroxylating the proline residues of HIF-α [58]. The enzymatic properties of the recombinant proteins were analyzed, and these enzymes were clearly identified as dioxygenases that hydroxylate proline or asparagine residues; they require molecular oxygen, 2-OG, Fe^2+^, and ascorbic acid as substrates [55,56,57] (Figure 2).

Notably, molecular oxygen is the substrate for these enzymatic reactions. The signal of a decrease in the partial pressure of oxygen as the substrate leads to a decrease of the activity of the oxygenase, resulting in a decrease in the ratio of hydroxylated HIF-α translated from the mRNA and an increase in the ratio of HIF-α recognized and bound by VHL [47]. VHL does not exhibit E3 ligase activity under hypoxia, and the ratio of ubiquitinated HIF-α decreases; consequently, the destruction of HIF is inhibited, and the dimer with HIF-1β protein is translocated to the nucleus where it acts as a transcription factor [59,60].

In their monumental paper published in 2001, Epstein et al. investigated the hydroxylation-modifying activity of HIF-1α using recombinant PHD1 protein generated in Escherichia coli through the in vitro transcription-translation method [15]. The activity of PHD1 has been shown to be suppressed in response to a change from 21% to 0% oxygen, simultaneously with the decrease in oxygen partial pressure.

Myllyharju et al. determined that the *Km* for PHD oxygen was 230–250 µM and that for FIH-1 was 90 µM, using a 20 amino-acid residue polypeptide as a substrate for a recombinant protein produced in insect cells. These results provide consistent evidence that PHD functions as an intracellular oxygen sensor, at least in the regulation of HIF-1 activity [58,61] (Table 1).

Decreased oxygen partial pressure leads to decreased VHL-mediated ubiquitination, resulting in a shift in the balance between protein destruction and translation, and a scheme or “dogma” of intracellular accumulation of HIF-1α protein [55].

## 7. Development of HIF-PHIs for Clinical Use

In the process of elucidating the molecular biological properties of HIFs, several small-molecule compounds have been observed to cause intracellular accumulation of HIF-α protein and increase transcriptional activity independent of the oxygen concentration or even under normoxic conditions [55,56,63]. Salts of Co^2+^, Cu^2+^, and Ni^2+^ were found to have hydroxylase inhibitory activity as antagonist of Fe^2+^ [38,39,40]. In addition, iron chelators such as deferoxamine mesylate, 3,4-dihydroxybenzoic acid, 1,10-phenanthroline, and quercetin were found exhibit enzyme inhibition [55,56,63]. These low molecular weight compounds have been found to activate HIF through the inhibition of prolyl and asparaginyl hydroxylases. However, these compounds are not specific HIF-α hydroxylase inhibitors. They also inhibit iron-dependent pathways in addition to HIF-α hydroxylase inhibition and may cause undue toxicity.

Characterization of the enzymes following the isolation of hydroxylase cDNA in 2001 has provided various insights into the effects of conventional HIF activators in terms of their effects on hydroxylase [55,57].

Dimethyloxalylglycine (DMOG), has been used as an HIF activator since early early research on HIF, primarily in basic experiments. DMOG is an antagonist of 2-OG, and it inhibits HIF-hydroxylases, including PHDs and FIH-1. Thus, the development and design of PHIs began with the synthesis of 2-OG analogs. N-oxalylglycine (NOG) was the first reported 2-OG mimetic molecule; however, DMOG, a precursor of NOG with the cell permeability of NOG, has been frequently used as a tool compound in research [63].

The synthesis of 2-OG analogs was the first approach employed in the design of PH inhibitors. Most molecules that have advanced to clinical use are 2-OG derivatives.

The active site-targeted inhibitors identified in this class exhibit strong binding interactions with hydrophobic residues in the 2-OG pocket. 2-OG mimics, such as NOG, allowed PHs and HIF binding, whereas larger heterocyclic inhibitors, such as FG-2216, stabilize the closed conformational structure and prevent binding of the substrate to the PHs [64,65]. Most PH inhibitors are composed of three structural features based on ligand-protein interactions. The first feature is a bidentate coordination site on the iron atom. The second important feature is a carboxylic acid that forms a salt bridge with the Arg383 side chain. The third attribute is a hydrogen bond acceptor for the phenolic hydroxyl of Tyr303 [55,63,66].

There are more than sixty 2-OG-dependent hydroxylases in the body; however, the inhibitors on the market are more than 1000 times more specific for PHD1-3 than for FIH-1, and the inhibition of HDACs and other enzymes is negligible [58].

**Figure 3:**
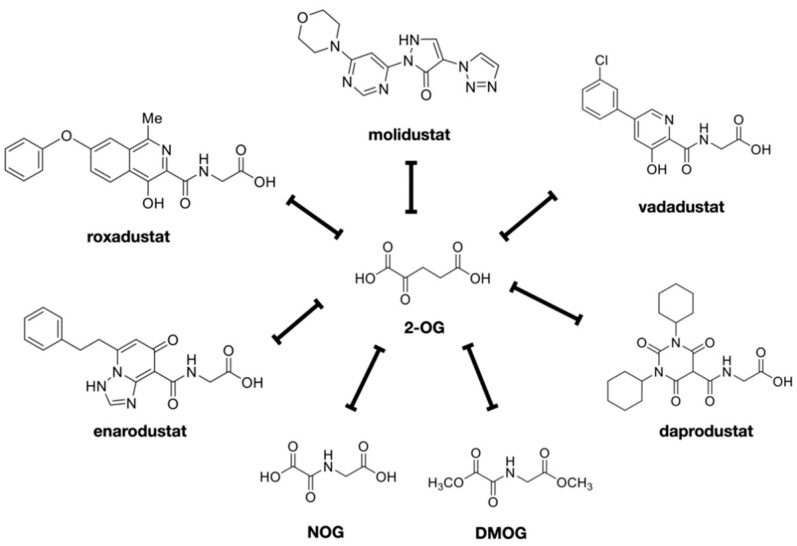
HIF-PHIs available in clinical field. HIF-PH inhibitors were shown. These are all competitive inhibitors of 2-OG. NOG and DMOG are the prototypes of these drugs. Daprodustat, enarodustat, molidustat, molidustat, and vadadustat are the inhibitors currently used in clinical medicine in Japan.

There are five HIF-PHIs that have completed Phase III trials and are now being marketed and prescribed in clinical practice in Japan: daprodustat, roxadustat, vadadustat, molidustat, and enarodustat (Figure 3). Desidustat (ZYAN1) and JNJ-429045343, which is under preclinical development, are new drugs under development. Although there are recognized class effects, each has a different molecular structure, half-life, and adverse event profile, and there is diversity in PHs selectivity.

There is also diversity in PHs selectivity. For example, molidustat mainly inhibits PHD2, daprodustat inhibits PHD2 and PHD3, while roxadustat seems to inhibit all three PHDs. It remains to be seen whether such selectivity affects the malignancy risk of individual PHDs. Among various drugs that have been developed, at least five HIF-PHIs, approved for the treatment of renal anemia in Japan, are being used in clinical practice as of 2021 [24].

The recombinant protein of HIF-PHs including PHD1-3 has become available, laying the foundation for the assay and development of inhibitors [58,67]. On the basis of the general assay for 2-OG-hydroxylase activity, various assays have been proposed and reported for HIF-hydroxylase activity [68], including assays based on 2-OG/O2 consumption and succinate/CO2 production [69,70,71] and cell-based assays including the classical manual method of detecting HIF-1α/2α proteins with specific antibodies and using HRE-reporters has been proposed [72]. Other cell-based assays are based on measuring changes in the expression of well-characterized HIF transcriptional targets, such as EPO. A recent advancement has been the development of HIF-1α hydroxylation site-specific antibodies, which allow semi-quantitative analysis of the relative efficiency of intracellular PHDs and FIH-1-catalyzed HIF-α hydroxylation [73,74,75,76,77,78,79].

In addition, cell-based assays have been developed. Additionally, the classical manual method of detecting HIF-1α/2α proteins with specific antibodies and using HRE-reporters has been proposed [72]. Other cell-based assays are based on measuring changes in the expression of well-characterized HIF transcriptional targets, such as EPO. A recent advancement has been the development of HIF-1α hydroxylation site-specific antibodies, which allow semi-quantitative analysis of the relative efficiency of intracellular PHDs and FIH-1-catalyzed HIF-α hydroxylation [73,74,75,76].

The hydroxyproline antibody-based amplified luminescent proximity homogeneous assay (AlphaScreen assay) was adapted to FG-4592 (roxadustat), GSK1278863 (dapro-dustat), vadadustat, and molidustat for measuring the PHD2-catalyzed hydroxylation of the HIF-1α peptide (HIF-1α residues 556–574). All “clinical” inhibitors potently inhibited the activity of PHD2, with IC_50_ values in the sub-micromolar range. Notably, molidustat (IC_50_ = 7 nM) is more potent than roxadustat (IC_50_ = 27 nM), vadadustat (IC_50_ = 29 nM), and daprodustat (IC_50_ = 67 nM), according to this assay [58]. To investigate the efficiency of inhibition of HIF-1α prolyl hydroxylation in cells, a luciferase-based hypoxia response element (HRE) reporter assay was performed with promoters containing tandem HRE sequences using established cell lines. All inhibitors strongly induced fire luciferase activity in a dose-dependent manner after 16 h of cell treatment, with EC_50_ values reported to be 5.1, 0.8, and 2.1 µM for roxadustat, daprodustat, and molidustat, respectively. In contrast, the ability of vadadustat (EC_50_ 41 µM, 16 h treatment) to induce HIF-1α in the HRE reporter assay was weaker than that of the other compounds specified above [58].

## 8. Metabolism of HIF-PHIs and Interactions with Other Drugs

HIF-PHIs are primarily eliminated through hepatic clearance, and drug interactions occur through the absorption and metabolism processes [80]. With the exception of daprodustat, concomitant use of oral drugs containing metal cations, polymeric phosphorus adsorbents, and iron agents decreases gastrointestinal absorption [81].

Several compounds have been screened as HIF-PHIs, and modifications have been made to develop compounds with good target enzyme inhibition ability, EPO production ability, cell permeability, oral absorption, and no accumulation in the body. Currently, several of these compounds are clinically used as drugs; however, there are certain differences in their pharmacokinetics. The pharmacokinetic characteristics of HIF-PH translation are summarized in the Table 2; hepatic metabolism involves CYPs and conjugating enzymes, transporters mediate transport in and out of hepatocytes, and renal interaction involves organic anion transporters (OATs).

With respect to metabolism, the effects of metabolic enzymes and transporter inhibitors are known; for example, the blood concentrations of roxadustat and daprodustat are increased by the concomitant use of CYP2C8 inhibitors. In addition, HIF-PHIs inhibit transporters in the liver and kidney, which may enhance the effects of concomitant drugs. Therefore, when applying HIF-PHIs, it is necessary to evaluate drug interactions occurring via these mechanisms (Table 2).

Roxadustat is taken up by organic anion transporting polypeptide (OATP) 1B1 in hepatocytes, hydroxylated by CYP2C8 or conjugated to a sulfate group, and glucuronosylated by uridine diphosphate glucuronosyltransferase (UGT) 1A9 [82,83]. Vadadustat is glucuronosylated by UGT1A1, and its contribution to metabolism is small. Solubility increases with increase in pH; however, no effect has been observed with the concomitant use of proton pump inhibitors [84]. Daprodustat is metabolized by CYP2C8, and its blood concentration increases with the concomitant use of CYP2C8 inhibitors [81]. The half-life of daprodustat is shorter than that of other HIF-PHIs. No effects of diet or phosphorus adsorbents were observed. Gemifibrozil, a CYp2C8 inhibitor, increased the AUC of daprodustat by 18.6 folds. Enarodustat is hardly sensitive to metabolism and is not affected by CYP or other factors [85]. It is primarily eliminated through hepatic clearance, excreted in the bile, and circulated in the intestinal tract; its apparent elimination half-life is relatively long [86]. Molidustat is primarily glucuronidated by UGT1A1, and approximately 85% of the administered dose is recovered in the urine as N-glucuronidated metabolites [87,88,89,90].

**Table 2:** Pharmacologic profiles of HIF-PHIs.

Drug	Absorption	Excretion Rate of Unchanged Substance in Urine	Half Life (h)	Major Metabolic Pathways	Ref.
Roxadustat	40~80%	1%	12~15	CYP2C8, UGT1A9	[82]
Vadadustat	>75%	<1%	4~7	UGT1A1/1A9	[84,91]
Dapurodustat	65%	<0.05%	1~7	CYP2C8	[81,92]
Enarodustat	41.70%	27~61%	~11	Less susceptible to metabolism	[85,93]
Molidustat	59%	4%	4~10	UGT1A1/1A9	[88,89]

## 9. Regulatory Mechanism of Erythropoiesis

EPO is a major external factor for erythropoiesis during the differentiation and maturation of erythroid lineage cells from hematopoietic stem cells [94]. EPO is secreted from the kidney by hypoxia, and EPO acts on the EPO receptor (EPOR) of erythroblast progenitor cells; EPOR is present in the plasma membrane as a homodimer. Signaling from EPO to EPOR includes the JAK2/STAT5-, PI3K/Akt-, and MAP kinase-mediated pathways. The JAK2/STAT5-mediated pathway is particularly important. When EPO binds to EPOR, the three-dimensional structure of EPOR changes, and the bound JAK2 is autophosphorylated. The phosphorylated JAK2 phosphorylates the tyrosine residue of EPOR, which causes the phosphorylation of STAT5, which is transferred to the nucleus; STAT5 acts as a transcription factor in cooperation with GATA-1 [95]. Signals from EPOR inhibit apoptosis and increase the expression of genes required for erythroid differentiation. EPOR promotes the proliferation of erythroleukemia cell lines and various tumor cells. In contrast to anemia, polycythemia involves an abnormal increase in the number of RBCs. Polycythemia vera and familial erythrocytosis are two types of polycythemia caused by genetic abnormalities. In polycythemia vera, the V617F mutation in the JAK2 gene in hematopoietic stem cells homeostatically activates cell proliferation signals, including the EPOR, leading to an increase in the number of cells in the three lineages erythrocytes, granulocytes, and platelets [95,96,97]. In addition to polycythemia vera, this mutation is often found in myeloproliferative tumors, such as primary myelofibrosis and essential thrombocythemia. Four types of familial erythrocytosis have additionally been reported. Type 1 is caused by a genetic abnormality of EPOR, resulting in increased reactivity to EPO and an increased erythrocyte count [98]. The serum EPO concentration was low. Type 2 is common in the Chuvash region of the Volga River basin in Russia, where the Turkic peoples live. It is caused by mutations in the VHL gene [99,100]. The mutant VHL protein is unable to bind to E3 ubiquitin ligase and HIF-1, which inhibits the degradation of HIFα and increases the expression of HIF target genes such as EPO, glucose transporters, transferrin, transferrin receptor 1 (TFR1), and VEGF. The Hb level can be over 20 g/dL; blood pressure is low; and the frequency of myocardial infarction, thrombosis, and spinal cord hemangioma is high. Unlike in type 1, serum EPO concentration is increased in type 2. Type 3 is caused by mutations in the PHD2 (*EGLN1*) gene [101,102]. Type 4 is caused by mutations in the HIF2A (*EPAS1*) gene; the mutant HIF-2α protein is not degraded, resulting in increased expression of EPO and erythrocytosis [103]. Additionally, serum EPO concentration increases. Thus, various reports indicate that activation of the HIF pathway can lead to improvement of anemia. Thus, the focus of research moved in the direction of artificially modifying PH activity.

## 10. Renal Anemia Due to CKD

Anemia caused primarily by renal failure is defined as renal anemia [104,105,106]. REP cells are localized in the interstitium of normal kidneys [43,107]. They are widely distributed from the outer layer of the tubulointerstitial medulla to the cortex, and they border interstitial capillaries [108,109]. Near the REP cells are tubular epithelial cells that consume large amounts of oxygen during urine reabsorption. Thus, the oxygen environment in the paratesticular capillaries is variable. Not all REP cells produce EPO uniformly, and some cells express EPO genes in a flexible and sensitive response to oxygen concentration [36]. HIF-2 is a regulator of this system. Under hypoxia, HIF induces EPO gene expression, whereas under normoxia, oxygen and proline hydroxylase bind to the HIF-2α protein, which is rapidly degraded and which does not stimulate EPO production in the REP cells. Interstitial fibrosis secondary to renal failure is a major pathomorphological finding in renal injury, and REP cells that are transformed from fibroblasts to myofibroblast-like cells play a major role in interstitial fibrosis. Myofibroblasts play a major role in fibrosis, and a study tracing the origin of renal myofibroblasts determined that half of them were derived from stromal fibroblasts, 35% from the bone marrow, 10% from endothelial cells, and 5% from epithelial cells [110,111,112]. In addition, studies in animal models have shown that many of the myofibroblasts are transformed from cells that originally exhibited the properties of REP cells; when REP cells are transformed, they strongly express α-smooth muscle actin, leading to progressive fibrosis. In the fibrotic area, capillaries around the tubules drop out; additionally, tissue oxygen and nucleic acids are disturbed.

Fibrosis further lowers the partial pressure of oxygen in the kidneys. However, although the partial pressure of oxygen is lowered, the transformed REP cells lose their ability to produce EPO [36]. Thus, at the stage of renal interstitial fibrosis, even if the oxygen concentration decreases, EPO production is insufficient, resulting in a lack of oxygenated hemoglobin; the remaining tubular cells do not receive enough oxygen because the tubules consume high amounts of oxygen. This is the pathophysiology of renal anemia [36].

Uremic substances induce oxidative stress and inflammatory cytokines; impair iron utilization via hepcidin; and affect HIF activation.

As of 2016, the number of patients with end-stage renal failure reached 3.73 million worldwide; this figure is increasing at a rate of 5–6% per year. This includes 2.65 million on hemodialysis, 340,000 on peritoneal dialysis, and 740,000 on renal transplantation, with all treatment modes increasing at a rate of 5–6% per year.

The frequency and severity of renal anemia worsens with the development of renal dysfunction.

Chronic anemia has been shown to exacerbate renal dysfunction, increase cardiovascular complications, and adversely affect life expectancy because of the development of organ damage due to ischemia, leading to the cardiac-renal-anemia syndrome.

## 11. HIF-PHIs as a Treatment for Renal Anemia

Absorption of orally administered HIF-PHIs from the intestinal tract can result in HIF activation in most cells in the body. HIF-PHIs are clinically used for the treatment of renal anemia [24,25,113].

Treatment of renal anemia improves quality of life, which is impaired due to poor exercise tolerance caused by chronic anemia; additionally, it has been shown to exhibit organ protective effects. For example, therapeutic intervention with ESA from the early stage of CKD, which has become a serious problem, is expected to not only improve the quality of life of the patients and avoid blood transfusion but also to improve life prognosis through organ protection, including prevention of progression of renal dysfunction.

When ESAs were introduced into the clinic in the 1980s, their effectiveness in improving anemia was confirmed, and several clinical studies were conducted primarily in Europe and the United States to establish safe and effective treatment guidelines [114]. However, contrary to expectations, there have been reports of increased adverse events such as cardiovascular complications or no improvement in prognosis when high hemoglobin levels were used as the target for correction [115]. However, the target Hb levels in these clinical trials were approximately 11 g/dL and 13 g/dL or higher; compared to Hb levels of approximately 11 g/dL, there was no additional prognostic effect even if the Hb levels were improved to a level equivalent to that of healthy subjects [115,116]. These results suggest that continued administration of high-dose ESAs to patients with a low hematopoietic response to ESAs may increase the risk of life-threatening events. In response to these results, Western countries, which had been aiming for normal Hb levels, took a turn and revised their targets downward. The KDIGO (The Kidney Disease Improving Global Outcomes) guideline published in 2012 states that with regard to the upper limit of targets for patients with conservative CKD, it is desirable not to administer ESAs to maintain Hb levels above 11.5 g/dL. If the Hb level is less than 10.0 g/dL, the decision to initiate ESA therapy should be individualized on the basis of the rate of decline in Hb concentration, response to prior iron therapy, risk of needing a blood transfusion, risks associated with ESA therapy, and the presence or absence of symptoms associated with anemia [117,118]. This does not mean that uniform criteria should be set. In addition, for HD patients, it was stated that it is desirable to start ESA therapy when the Hb level does not fall below 9.0 g/dL, as it is not reasonable to maintain Hb levels above 11.5 g/dL [116]. It is now advocated that renal anemia should be managed with adequate iron supplementation followed by ESA or HIF-PHIs [24,116]. The target hemoglobin level for CKD in the conservative phase is 11–13 g/dL and that for CKD in the hemodialysis phase is 10–12 g/dL. The target Hb level was determined and treated according to the pathology of each individual case [24,116].

As mentioned above, the canonical target of HIF-PHIs for EPO production is the renal interstitial REP cells, which stabilize HIF-1α and HIF-2α and induce endogenous EPO expression to promote erythropoiesis [119,120]. HIF-PHIs have been reported to increase EPO production in patients undergoing hemodialysis even after the removal of both kidneys, and PHD1-3 inhibition in hepatocytes restores EPO production, suggesting that EPO production in organs other than the kidney, including the liver, may contribute to the improvement of anemia.

**Figure 4:**
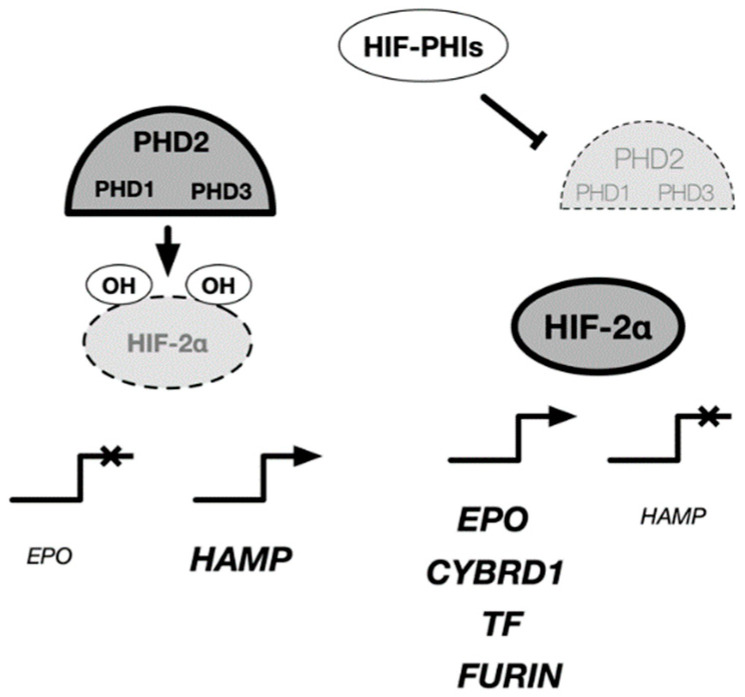
Regulatory mechanism of EPO and iron metabolism-related genes in cells. HIF-PH inhibitors have been prescribed for the treatment of renal anemia. The target cells of HIF-PH inhibitors include Rep cells located in the kidney, liver, and intestinal epithelial cells, where activation of HIF-2 rather than HIF-1 induces expression of various genes related to EPO and iron metabolism. In the liver, HIF-2 is known to have an inhibitory effect on hepcidin expression. EPO: erythropoietin, HAMP: hepcidin, CYBRD1: Dcytb, TF:transferin, FURIN: furin.

HIF-PHIs may additionally contribute to the improvement of anemia by improving iron metabolism. Hepcidin, a peptide hormone produced in the liver, plays a central role in regulating iron metabolism by turning over and recycling iron in the body inhibitors. The iron-supply mechanism includes transferrin receptor 2 (TFR2), hepcidin/ferroportin (FPN), and iron regulatory protein (IRP), which sense changes in intracellular iron concentration. IRP creates a feedback mechanism that ultimately maintains a constant level of serum iron and hemoglobin. During evolution, iron was a difficult element to ingest, and humans were believed to have been iron starved. Therefore, humans do not have a system for excreting iron. The iron required by the body is absorbed through the intestinal tract. Orally ingested inorganic iron is taken into the body via DMT1 (divalent metal transporter 1) in the duodenal epithelium, while heme iron is taken into the body via HCP-1 (heme carrier protein 1). The expression of DMT1 and FPN is regulated by IRP through sensing the amount of intracellular divalent iron. As the iron concentration increases during absorption (mucosal uptake), DMT1 expression decreases, and new absorption is restricted. In contrast, mucosal transfer of iron into the blood is mediated by FPN, which is regulated by hepcidin. As serum hepcidin levels increase, intestinal iron absorption decreases. In renal anemia, oral iron is believed to be ineffective, and injections should be utilized; the cause of this ineffectiveness is increased serum hepcidin levels (Figure 4).

In CDK patients, hepcidin levels are elevated due to decreased excretion and chronic inflammation. Hepcidin internalizes and degrades ferroportin, the only iron efflux protein present in vivo, thereby inhibiting iron absorption from the intestinal epithelium, iron release from hepatocytes, and iron cycling from macrophages. This results in increased serum iron concentration and intracellular iron and impaired iron utilization in the bone marrow.

In addition, transferrin and transferrin receptors, which are involved in iron transport, are target genes of HIF-1, and the expression of DMT1 and Dcytb, which are involved in intestinal iron absorption, are regulated by HIF-2 (Table 3).

In particular, it has been reported that hepcidin production is increased under inflammatory conditions, causing ESA resistance because iron stores are not available for hematopoiesis.

Previous clinical trials have shown that patients with high CRP require larger doses of ESAs to maintain the same Hb level than those with low CRP levels; however, there is no difference in the dose of roxadustat required, regardless of the CRP level.

Regarding the effect of iron metabolism, the required dose of ESAs was reported to be higher in iron-deficient patients with low TSAT and ferritin levels than that in iron-deficient patients with TSAT and ferritin levels above the guideline recommendations; in contrast, the difference in dosing was minor with roxadustat [133,134,135,136].

Thus, HIF-PH inhibitors may contribute to the improvement of anemia by stimulating EPO production and altering iron metabolism in favor of hematopoiesis.

## 12. Nephroprotective Effects of HIF-PHIs

HIF-PHIs are expected to be effective in treating other diseases and conditions, including acute kidney injury (AKI) and renal transplantation [137,138].

In AKI, hypoxia caused by renal ischemia leads to functional decline of tubular epithelial cells, resulting in degeneration and necrosis [139,140,141,142]. Urothelial epithelial cells are oxygen-consuming cells with abundant mitochondria that require a large amount of oxygen for the reabsorption and secretion of substances [143]. This indicates that they are vulnerable to hypoxia [144,145,146].

In kidney transplantation, artificial ischemic AKI occurs during the removal of the transplanted kidney from the donor. After transplantation into the recipient, the resumption of blood flow causes ischemia-reperfusion injury to the transplanted kidney [147,148,149,150].

AKI and renal ischemia-reperfusion cause various physiological changes, including oxidative stress updating, inflammatory cytokine release, mitochondrial dysfunction, autophagic dysfunction, and hyperglycemia. During the anastomosis in kidney transplantation procedure, the transplanted kidney from the donor was maintained at a low temperature.

AKI in the kidney induces activation of HIFs in tubular epithelial cells and renal vasculature. This is believed to be a biological defense response.

HIFs have been reported to act as a protective mechanism for tubular epithelial cells in AKI by decreasing ROS and retaining glycogen [151].

Theoretically, HIF-PHI may protect against AKI or renal ischemia-reperfusion by activating HIF [152,153]. Cisplatin nephropathy in mice induced by roxadustat resulted in apoptosis and suppression of pro-inflammatory cytokines, indicating nephroprotection. In a report on the effect of the HIF-PHI roxadustat on AKI in cisplatin nephropathy [154], HIF-PHI improved renal function based on KIM-1 and NGAL levels as well as HIF-1 activation, suggesting that roxadustat induces profound changes in renal metabolism [155]. It is suggested that enalodustat may exert nephroprotective effects by altering energy-related metabolic metabolism in tubular cells [151].

In a rat ischemia-reperfusion model, the group pre-treated with daprodustat showed less renal interstitial fibrosis and interstitial inflammatory response after ischemia-reperfusion and less anemia deterioration than the control group [156]. FG-4497 administration to transplant donor rats improved the prognosis of transplanted grafts; upregulation of AgtPI4 and HO-1, inhibition of apoptosis, and activation of antioxidant pathways were observed in the renal tubular epithelium of the grafts [156].

These experimental results suggest that HIF-PHIs, when administered to renal transplant recipients or donors in advance, are promising nephroprotective agents against transient AKI or renal ischemia-reperfusion injury that occurs after renal transplantation. Renal expression of HIF has been reported to be associated with renal transplant rejection.

It may be used as a diagnostic pathological marker for rejection or as a prognostic marker for grafts.

## 13. Diverse Effects of HIF-PHIs

HIF-PHIs constitute a treatment for renal anemia with a completely new mechanism of action. Unlike conventional ESAs, which are injectable drugs that act specifically on the hematopoietic system, HIF-PHIs are oral drugs that may also have systemic effects. However, there is a concern regarding the possibility of side effects caused by the activation of defense mechanisms by HIF in conditions where angiogenesis plays a role in disease progression, such as cancer and retinal diseases. In addition to these theoretical concerns, adverse events have been reported in clinical trials.

### 13.1. Ischemia

Organ ischemia, or reduced blood flow, is a major clinical problem caused by diseases of the circulatory system. These ischemia may occur artificially as an interruption or bypass of circulation due to the needs of the surgical technique during surgery. All organs of the body can be affected, but in particular, the ischemia of brain, heart, kidneys, and extremities are the most clinically problematic. Ischemia can be acute in time (sudden decrease in blood flow within minutes to hours) or chronic (gradual decrease in blood flow over weeks to years). Tissue hypoxia is a common feature of ischemia, but the condition is complicated by decreased supply and discharge of metabolites. In these conditions, activation of the HIF system occurs, but the extent of this effect varies within and between ischemic tissues. Administration of HIF-PHI under these conditions is expected to increase HIF activity at a time that is precipitated by a decrease in blood flow, thereby enhancing endogenous defense and repair responses.

Multiple studies have been conducted to determine whether exogenous activation of HIFs can improve the outcome of experimental ischemia in various animal models [22,157,158,159,160]. The results of the majority of these studies suggest that HIF activation leads to improvement in pathology, at least in the short term. In models of cerebral ischemia, treatment immediately before or immediately after arterial occlusion has improved outcomes as assessed by infarct volume. Studies of coronary artery ligation have demonstrated the benefits of HIF-PHI administration, both in terms of reduction in infarct size and (when treated after ischemia) improvement in ventricular function [161,162,163].

Overall, a great number of ischemic protective mechanisms have been attributed to specific HIF target genes. These include genes involved in reprogramming cellular metabolism, affecting apoptosis/survival pathways, and altering vascular permeability. Others act on a longer time scale, such as angiogenesis and reperfusion, tissue repair, stem cell activation and homing, and matrix remodeling. However, it is still largely unknown which of these mechanisms mediate the effects observed in different ischemic models.

### 13.2. Inflammation

Inflammation is triggered in many diseases and plays an important role in the progression of the disease. Inflammation is caused by multiple factors, including responses to pathogens, tissue damage, and immune dysregulation, and is essentially a progressive condition. The high cytokine and chemokine environment and hypoxia induced by inflammation together lead to the activation of HIFs, which in turn have multiple effects on immune and inflammatory cells, including differentiation, apoptosis, and effects on cytokine production. Under a variety of circumstances, induction of HIF-1 has been reported to activate pro-inflammatory Th17 T cells by upregulating and co-activating the transcription factor ROR-γt and activating anti-inflammatory agents [164]. For example, activation of HIF may promote enhanced barrier function [165] and epithelial-mesenchymal transition [166] under various circumstances. On the other hand, however, artificial activation of HIF has been reported to improve the prognosis of various inflammatory models.

In an ulcerative colitis model in mice induced by administration of trinitrobenzene sulfonic acid, treatment with the HIF-PH inhibitors FG-4497 and AKB-4924 resulted in therapeutic effects based on weight loss, colon shortening, and reduced histological injury [160,167,168]. Mechanisms for this protective effect were proposed, including HIF-1-mediated improvement of epithelial barrier function and healing, and reduction of the inflammatory response. In another study of acute lung injury in mice, FG-4497 resulted in a decrease in leukocyte infiltration and was associated with increased survival of individuals [169]. The mechanism of protection in this study was proposed to be HIF-2-dependent induction of the vascular endothelial protein tyrosine phosphatase, leading to an increase in the integrity of the vascular barrier in the lung. Some studies have examined the effects of HIF-PHI on inflammation associated with bacterial infections; GSK360A was reported to alleviate elevated blood lactate levels and increase survival in a mouse model of endotoxin shock. It has been proposed that this effect is mediated by inhibition of the Cori cycle in the liver [170].

## 14. Adverse Effects of HIF-PHIs

### 14.1. Iron Deficiency

HIF-PH inhibitors cause a decrease in ferritin and an increase in TIBC after administration due to their complete effect on iron metabolism; administration of HIF-PHIs in iron-deficient patients may lead to iron deficiency, which reduces the effect of iron deficiency on anemia and causes iron deficiency symptoms in the bones, skin, and mucous membranes.

### 14.2. Cancers and Malignant Tumors

Clinical and animal studies of HIF-PH inhibitors have provided no evidence that HIF-PH inhibitors increase the incidence of renal cancer or other malignancies [90,119,120,133,134,135]. However, it cannot be denied that HIF activation by HIF-PH inhibitors may promote the proliferation, invasion, and metastatic potential of cells that have already undergone malignant transformation. It is important to note that HIF-1, but not HIF-2, is associated with the activity of malignant genes in tumors, and that activation of HIFs, particularly HIF-1, appears to be associated with the spread of metastases in breast, prostate, lung, bone, and colorectal cancers [171]. Nevertheless, HIF-2 has also been identified in malignant hepatocellular cell lines in vitro and is involved in the activation of cancer stem cell factors and is strongly associated with metastasis and even poor prognosis of various tumors [172]. In most kidney cancers, the VHL tumor suppressor gene is mutated or inactivated, resulting in the activation of the transcription factor HIF and upregulation of multiple genes downstream of HIF that are involved in cancer cell proliferation, invasion, and metastasis. In addition to renal cancer, increased expression of the HIF-1α protein has been positively correlated with cancer progression and metastasis in several solid tumors. The malignant transformation of cells requires the accumulation of driver gene mutations that play a decisive role in this process. Currently, there is no evidence that HIF-PHIs promote this process [173]. However, the effects of HIF activation on events such as mitosis, metastasis, and migration of cancerous cells are well predicted for the reasons described above. In addition, patients with the highest risk of kidney disease should be evaluated and followed up with appropriate imaging studies, such as MRI, contrast-enhanced CT, and ultrasound, before and after HIF-PHIs are administered and at least once a year after administration.

Nevertheless, normalization of tumor blood vessels is considered a useful therapeutic strategy to improve the tumor tissue environment [24].

On the basis of these findings, we investigated whether HIF-PHIs induce tumor vascular normalization and contribute to the improvement of the tumor tissue environment and found that administration of HIF-PHIs altered the vascular structure in tumor tissue [174]. In particular, fluorescent immunostaining of tumor tissues using CD31, a marker of vascular endothelial cells, showed that CD31 positivity per unit area of tumor tissue sections increased significantly after treatment with HIF-PHIs. A significant increase was observed in the evaluation of vessel length. A decrease in the number of vessels per unit area was observed during the evaluation of vessel density in the tumor tissue. These results suggest that HIF-PHIs suppress irregular branching and prolong the length of a single blood vessel in the tissue, reducing the specific characteristics of tumor vessels, such as irregular meandering and branching [174]. In addition, extravascular leakage was reduced in HIF-PHI-treated tissues. These results suggest that HIF-PHIs not only induce structural changes in blood vessels but also functionally improve normal blood vessels. These results suggest that HIF-PHI-induced vascular normalization improves the drug delivery efficiency of anticancer drugs and enhances anticancer drug sensitivity. In addition, HIF-PHIs have been reported to restore the immune responsiveness of immune cells in tumors by improving the tumor-specific tissue environment, resulting in therapeutic effects mediated by immune cells [175].

### 14.3. Diabetic Retinopathy and Age-Related Macular Degeneration

HIF-1α and VEGF induced by HIF-1α are closely related to the development and progression of diabetic retinopathy and age-related macular degeneration (AMD). HIF-PHIs may increase the expression of VEGF and angiogenesis through the activation of HIF. There are concerns that stabilization of HIF may exacerbate retinal lesions, especially diabetic retinopathy, which is a common complication in dialysis patients. However, no clinical study reported to date has been associated with worsening of retinopathy, and the results of clinical trials indicate that retinal hemorrhage was as frequent in the HIF-PHI group as in ESA group for all drugs [90,119,120,133,134,135]. However, it has been suggested that VEGF may be expressed locally in the retina and that it may act in an autocrine and paracrine manner; EPO itself is suggested to be associated with retinopathy. In addition, several clinical trials have excluded patients at high risk of retinal hemorrhage; therefore, caution should be exercised during the use of HIF-PHIs to ensure that retinopathy does not worsen, especially in patients who already have retinopathy.

### 14.4. Thromboembolism

Since thromboembolism can be caused by a sudden increase in the viscosity of the blood, the rate of increase in hemoglobin levels should not exceed 0.5 g/dL/week. To this end, the dose of HIF-PHIs should be increased gradually, with appropriate intervals between increases, in accordance with the label of the respective drug [25,176]. Since iron deficiency itself has been reported to be a risk factor for thromboembolism, iron deficiency should be avoided [25,177,178]. Signs and symptoms of suspected thromboembolism should be closely monitored during treatment. Specific suspected symptoms include significant left-right difference in leg edema (suspicion of deep vein thrombosis), poor bleeding out of the vascular access (precursor of vascular access occlusion), transient ischemic attack (precursor of cerebral infarction), and sudden onset of rapid loss of vision or blurred vision (suspicion of retinal vein occlusion). If these symptoms occur, in addition to the evaluation of hemoglobin level, FDP, vascular access echocardiography, brain MRI, and ophthalmologic evaluation should be performed as soon as possible, and immediate action should be taken. If these symptoms clearly increase after initiation of HIF-PHIs, the inhibitor treatment should be discontinued.

HIF-1 activation by hypoxia promotes vascular calcification by transforming smooth muscle cells into osteoblast-like cells, and some CKD patients exhibit significant vascular calcification; therefore, care should be taken when administering HIF-PHIs [179].

### 14.5. Pulmonary Hypertension

Studies using patients with mutations in HIF-related genes and animal models have indicated that constant activation of HIF signaling may exacerbate pulmonary hypertension [180]. Considering the characteristics of the pulmonary vascular response to oxygenation, the risk of developing pulmonary hypertension due to administration of HIF-PHIs should be carefully considered. When HIF-PHIs are administered, patients should be carefully interviewed to ensure that exercise tolerance is not impaired. In addition, electrocardiography and echocardiography should be performed periodically to check for right ventricular strain findings. In particular, if peak tricuspid regurgitation velocities of 3.4 m or more per second occur, referral to a cardiologist should be considered. The use of HIF-PH inhibitors in patients who have already developed pulmonary hypertension should be considered with extreme caution. Although the relationship between HIF-PHIs and cardiovascular events in Japanese patients is unclear at this time, in animal studies, constant activation of HIF signaling has been reported to cause heart failure [181,182,183,184,185,186,187,188]. When HIF-PHIs are administered, it is recommended that chest radiography and echocardiography be performed periodically. It should be noted that the BNP levels in the blood may be directly induced by the administration of HIF-PHIs, as well as by renal function [189].

### 14.6. Polycystic Kidney Disease (PCKD)

HIF-1α has been reported to be involved in the enlargement of renal scales in an animal model of advanced polycystic kidney disease [190]. In contrast, there was no increase in the number of renal scales in a mild animal model of polycystic kidney disease [191]. It is not known whether HIF-PHIs induce further HIF activation in patients with multiple cysts; local HIF activation in the kidney has been reported to occur because of reduced blood flow caused by compression by the polycysts. However, because of the short duration of the trial, further long-term evaluation is necessary to determine the effect.

The development of hepatic dysfunction is a significant issue; in fact, the HIF-PHI FG-2216, which was clinically developed in 2008, was withdrawn from the market owing to the development of fatal liver failure in one patient.

HIF has been reported to be associated with hepatic fibrosis and inflammation in nonalcoholic fatty liver disease; therefore, abnormal liver function should be closely monitored [192,193,194].

### 14.7. Hyperkalemia

A phase III clinical trial in China reported a significant increase in hyperkalemia and metabolic acidosis complications in both conservative CKD patients and hemodialysis patients treated with roxadustat [119,120]. Additionally, daprodustat clinical trials reported a significant increase in hyperkalemia, as a side effect, in hemodialysis patients [195]. We cannot rule out the possibility that this side effect is a class effect of HIF-PHI. However, the incidence of hyperkalemia was comparable between the roxadustat and control groups when analyzed using data. In addition, several studies have reported a higher incidence of hyperkalemia in the HIF-PHIs group, but definitive conclusions about this side effect have not yet been reached. Considering that hyperkalemia is a common and life-threatening complication, especially in patients with kidney disease, it is necessary to monitor serum potassium regularly after initiation of HIF-PHIs as well as during treatment.

## 15. Establishment of Resistance

Clearly, our body has a built-in negative feedback mechanism to prevent HIF from being constantly activated. The HIF system forms a negative feedback loop [196,197]. HIF-1 activation has been observed to repress HIF-dependent gene responses through the induction of PHD2 and PHD3 mRNA expression [198,199].

HIF-PHIs activate HIF and may induce the mRNA and protein expression of PHD2 and PHD3 independent of the oxygen concentration. If HIF-PHIs trigger this feedback loop and alter EPO induction and iron metabolism-related gene expression, long-term use may lead to resistance against HIF-PHIs.

## 16. Conclusions

HIF-PHIs activate HIF-1 and HIF-2 by exogenously triggering the hypoxia-responsive gene response in the body. It has been shown that HIF activation stimulates EPO production by REP cells and improves the oxygen-carrying capacity of the blood through proliferation of erythrocytes by increasing the efficiency of iron metabolism. Currently, five orally administered HIF-PHIs are available for the treatment of renal anemia in clinical practice. It is necessary to continue to carefully study their adverse effects on malignancy and retinopathy and the currently unknown effects of their long-term use.

## Figures and Tables

**Table 1:** Enzymatic properties of HIF-α prolyl and asparaginyl hydroxylases [61,62]

Substrate				*Km* (µM)			
Gene Name	Enzyme	Pro-402	Pro-564	O_2_	2-OG	Ascorbate	Fe (II)
*EGLN1*	PHD2	+	+	230	60	170	0.03
*EGLN2*	PHD1	+	+	250	60	180	0.1
*EGLN3*	PHD3	−	+	230	55	140	0.03
*HIF1AN*	FIH-1	Asp-803		90	25	260	0.5

**Table 3:** HIFs target genes involved in iron homeostasis.

Protein	Gene	HIF1/HIF2	Function	Ref.
Ceruloplasmin	*CP*	HIF-1	ferroxidase	[121]
Duodenal cytochrome b	*CYBRD1*	HIF-2	Ferric reductase	[122]
Erythropoietin	*EPO*	HIF-2	promote red blood cell production	[36,123]
Ferrochelatase	*FECH*	HIF-1	Heme synthesis	[124]
Furin	*FURIN*	HIF-1	subtilisin-like proprotein convertase	[125,126,127]
Hepcidin	*HAMP*	HIF-2	maintenance of iron homeostasis	[128,129]
Heme oxygenase-1	*HMOX1*	HIF-1	Heme degradation	[121]
Aconitase	*IRP1*	HIF-1	Cellular iron sensing	[130]
Transferrin	*TF*	HIF-1	Serum iron transfporter	[131,132]
Transferrin receptor	*TFRC*	HIF-1	Cellular iron uptake	[121]

## Data Availability

This study did not report any data.
